# Attention mechanism models for precision medicine

**DOI:** 10.1093/bib/bbae156

**Published:** 2024-05-29

**Authors:** Liang Cheng

**Keywords:** attention mechanism model, precision medicine

## Abstract

The development of deep learning models plays a crucial role in advancing precision medicine. These models enable personalized medical treatments and interventions based on the unique genetic, environmental and lifestyle factors of individual patients, and the promotion of precision medicine is achieved mainly through genomic data analysis, variant annotation and interpretation, pharmacogenomics research, biomarker discovery, disease typing, clinical decision support and disease mechanism interpretation. Extensive research has been conducted to address precision medicine challenges using attention mechanism models such as SAN, GAT and transformers. Especially, the recent popularity of ChatGPT has significantly propelled the application of this model type to a new height. Therefore, I propose a Special Issue for Briefings in Bioinformatics about the topic ‘Attention Mechanism Models for Precision Medicine’. This Special Issue aims to provide a comprehensive overview and presentation of innovative researches on the application of graph attention mechanism models in precision medicine.

## REVIEWING THE PROGRESS OF ATTENTION MECHANISM MODELS IN PRECISION MEDICINE

Attention mechanism models have emerged as a powerful tool in precision medicine, enabling the integration and analysis of complex multimodal data. With the rapid advancement of high-throughput technologies, such as genomics, proteomics, metabolomics and clinical data, attention-based approaches have revolutionized our understanding of disease mechanisms and personalized treatment strategies.

Attention mechanisms and derived models have gained significant traction in drug development because of their outstanding performance and interpretability in handling complex data structures. Zhang *et al*. [1] offer an in-depth exploration of the principles underlying attention-based models and their advantages in drug discovery. The authors further elaborate on their applications in various aspects of drug development, from molecular screening and target binding to property prediction and molecule generation. Finally, they discuss the current challenges faced in attention mechanisms and artificial intelligence (AI) technologies, including data quality, model interpretability and computational resource constraints, along with future directions for research. In brief, the attention-based models will usher in revolutionary breakthroughs in the pharmaceutical domain, significantly accelerating the pace of drug development.

## INTRODUCING NOVEL ATTENTION MECHANISM MODELS FOR TUMOR BIOMARKER DETECTION RESEARCHES

Tumor biomarker detection plays a critical role in cancer research and clinical practice, enabling early diagnosis, prognosis prediction and targeted therapy. In recent years, attention mechanism models have emerged as innovative tools in the field of tumor biomarker detection, offering new avenues for analyzing large-scale multi-omics data and identifying crucial molecular signatures.

Jia *et al*. [2] discuss the limitations of spatial transcriptomics because of its high cost and propose an alternative approach using AI to predict spatial gene expression from histological images. Existing methods struggle to extract deep-level information from pathological images, prompting the development of THItoGene, a hybrid neural network that combines dynamic convolutional and capsule networks. This network aims to identify potential molecular signals in histological images and explore the relationship between pathology image phenotypes and gene expression regulation. The performance of THItoGene was evaluated using data sets from human breast cancer and cutaneous squamous cell carcinoma, demonstrating superior spatial gene expression prediction compared with existing methods. Additionally, THItoGene has shown its ability to decipher the spatial context and enrichment signals within specific tissue regions. The THItoGene tool is freely available on GitHub at https://github.com/yrjia1015/THItoGene.

Feng *et al*. [3] discuss the challenges presented by single-cell RNA sequencing (scRNA-seq) data, including heterogeneity, sparsity and complexity, and the need for more efficient and accurate machine learning models. The proposed approach, scMPN, integrates multilayer perceptron and graph neural network techniques, including an attention network, to perform gene imputation and cell clustering tasks. Evaluation of scMPN’s gene imputation performance is done using metrics such as cosine similarity, median L1 distance and root mean square error. Cell clustering efficacy is assessed using criteria like adjusted mutual information, normalized mutual information and integrity score. Experimental findings from four data sets with gold-standard cell labels demonstrate the superiority of scMPN over existing single-cell data processing techniques in both cell clustering and gene imputation investigations.

Wang *et al*. [4] argue that how to use existing data to improve the clustering quality of new scRNA-seq data remains an urgent challenge and it is critical to explore how information from different batches of RNA-seq data can be used to enhance comparability between data sets. To address these challenges, they developed a new method called graph-based deep embedding clustering (GDEC), which aims to cluster single-cell RNA-seq data from different species and batches. GDEC combines Graph Convolutional networks and deep embedding clustering to gain important prior knowledge from existing data sets to better understand gene expression patterns and cellular relationships between different cell types. GDEC could effectively realize cross-species and cross-batch scRNA-seq data clustering. The validity of GDEC was validated by comparing with Seurat, Moana and scVI models—performing better in finer-grained cell classification, prediction of unknown cell type and detection of changes in cell gene expression. These results highlight the effectiveness of the suggested methodology in leveraging deep learning approaches to enhance the interpretation of scRNA-seq data.

## EXPLORING NOVEL NATURAL LANGUAGE PROCESSING MODELS FOR TARGETED DRUG DESIGN AND CLINICAL DIAGNOSIS

Natural language processing (NLP) has emerged as a promising approach in the field of drug design and clinical diagnosis, enabling efficient analysis and interpretation of complex biomedical data. With recent advances in deep learning and neural network architectures, novel NLP models have been developed that can effectively leverage vast amounts of text-based information and provide valuable insights into disease pathogenesis, drug discovery and personalized treatment.

Li *et al*. [5] study aims to develop a data-driven predictive model for portal vein thrombosis (PVT) diagnosis in chronic hepatitis liver cirrhosis patients. They employ data from a total of 816 chronic cirrhosis patients with PVT, encompassing a wide range of variables, including general characteristics, blood parameters, ultrasonography findings and cirrhosis grading, divided into the Lanzhou cohort (*n* = 468) for training and the Jilin cohort (*n* = 348) for validation. To build a predictive model, the authors employ a sophisticated stacking approach, which includes support vector machine (SVM), Naïve Bayes and quadratic discriminant analysis (QDA). Comparative analysis shows that their QDA model outperformed several other machine learning methods. Their study presents a comprehensive data-driven model for PVT diagnosis in cirrhotic patients, enhancing clinical decision-making. The SVM-Naïve Bayes-QDA model offers a precise approach to managing PVT in this population.

Pham *et al*. [6] introduce H2Opred, a novel hybrid deep learning model specifically designed to accurately identify 2OM sites in human RNA. H2Opred utilizes stacked 1D convolutional neural network (1D-CNN) blocks and stacked attention-based bidirectional gated recurrent unit (Bi-GRU-Att) blocks. The 1D-CNN blocks learn feature representations from conventional descriptors, whereas the Bi-GRU-Att blocks extract feature representations from NLP-based embeddings derived from RNA sequences. H2Opred combines these representations for accurate predictions. Extensive cross-validation analysis demonstrates that H2Opred consistently outperforms ML-based single-feature models on five different data sets. The generic model of H2Opred shows remarkable performance on both training and testing data sets, surpassing existing predictors and other nucleotide-specific models. To enhance accessibility, a user-friendly web server for H2Opred has been deployed (https://balalab-skku.org/H2Opred/).

Li *et al*. [7] discuss the challenges of multi-omics data integration in biomedical research and the increasing popularity of consensus clustering as a tool for phenotyping and endotyping using multiple omics and clinical data. However, current consensus clustering methods rely on ensembling clustering outputs with similar sample coverages, which may not reflect real-world data with varying sample coverages. To address this issue, the authors propose a new consensus clustering with missing labels (ccml) strategy, an R protocol that can handle unequal missing labels. The ccml method was applied to predict distinct groups based on omics integration in two different cohorts investigating chronic obstructive pulmonary disease and adult asthma patients. The authors propose ccml as a downstream toolkit for multi-omics integration analysis algorithms to overcome limitations posed by missing data.

## IDENTIFYING DISEASE MECHANISMS RELATED TO MAJOR CHRONIC DISEASES

Chronic diseases are responsible for a significant burden of morbidity and mortality worldwide. Despite extensive research efforts, the etiology and mechanisms underlying these diseases remain poorly understood. However, recent advances in genomics, proteomics, metabolomics and other omics-based technologies, coupled with sophisticated computational approaches, have provided unprecedented opportunities to identify disease mechanisms and potential therapeutic targets.

Noncoding RNAs (ncRNAs) occupy a significant portion of the human genome and participate in gene expression regulation through various mechanisms. Several recent studies have shown that ncRNAs play a crucial regulatory role in gestational diabetes mellitus (GDM). Here, Gao *et al*. [8] present a comprehensive review of the multiple mechanisms of ncRNAs in GDM along with their potential role as biomarkers. In addition, the authors investigate the contribution of deep learning-based models in discovering disease-specific ncRNA biomarkers and elucidate the underlying mechanisms of ncRNA. This review will assist community-wide efforts to obtain insights into the regulatory mechanisms of ncRNAs in disease and guide a novel approach for early diagnosis and treatment of disease.

In conclusion, I hope this Special Issue will be helpful to the attention mechanism models for precision medicine and provide inspirations for the mechanisms of disease. I would like to express my sincere thanks to contributing authors for their excellent efforts.

## Data Availability

No public data has been usedand no new data has been generated in this article.

## Author Biographies

**Liang Cheng** is a professor from Harbin Medical University in Harbin, Heilongjiang Province, China, focusing on machine learning algorithms, multi-omics data processing, and gut microbiome research.
